# In Vitro Starch Digestibility, Rheological, and Physicochemical Properties of Water Caltrop Starch Modified with Cycled Heat-Moisture Treatment

**DOI:** 10.3390/foods10081687

**Published:** 2021-07-21

**Authors:** Po-Ching Tsai, Lih-Shiuh Lai

**Affiliations:** Department of Food Science and Biotechnology, National Chung Hsing University, 145 Xingda Road, Taichung 40227, Taiwan; ss40616ss@gmail.com

**Keywords:** starch modification, rheological properties, predicted glycemic index

## Abstract

This study focused on the effect of cycled heat-moisture treatment (cHMT) on the in vitro digestibility, rheological, and physicochemical properties of water caltrop starch. The amylose content increased significantly by cHMT, whereas damaged starch content decreased only in the groups with more than two cycles applications. cHMT generally increased the weight-average molecular weight, except for single cycle treatment which showed the reverse result. In thermal properties, the onset temperature (T_0_), peak temperature (T_p_), and conclusion temperature (T_c_) increased, while the enthalpy needed to complete the gelatinization was lowered by cHMT. Water caltrop starch paste showed less shear-thinning behavior with cHMT. Meanwhile, the viscosity and tendency to form strong gel were enfeebled with modification. cHMT significantly changed predicted glycemic index (pGI) value, especially in samples that underwent the most cycles of treatment, which showed the lowest pGI compared to native and other treatment. These results suggested that cHMT water caltrop starch was effectively modified and showed diversified properties.

## 1. Introduction

Water caltrops (*Trapa taiwanensis* Nakai) are aquatic crops cultivated in shallow water ponds especially in Southern Taiwan. The kernels are mainly composed of starch and can reach a maximum content of 79.4% (d.b.) during growth [1,2]. Water caltrop starch showed higher onset temperature (T_0_), peak temperature (T_p_), and conclusion temperature (T_c_) than corn and potato starch [3] and four aquatic vegetables in China [4]. It also showed solid-gel characteristics and dynamic rheological properties [5]. In other research, water caltrop also showed a great amount of resistant starch and a number of nutritional benefits; hence, water caltrops have been cultivated for years as food or traditional medicine in India and China [6,7].

Starch is a widely used material in the food industry. For example, it is used as a stabilizer [8], fat replacer [9], or versatile additive [10] in yogurt, baked goods, instant soups, sauces, and meat sausages [11]. However, native starch has some limitations in application due to its low heat stability, low shear resistance, and tendency to retrogradate. Starch has an essential role in nourishment and daily supplements of energy. According to the rate of digestion and glucose content released, predicted glycemic index (pGI) can be acquired by dividing the incremental postprandial blood glucose released from the samples by that of reference sample. Generally, lower GI is seen as a positive for patients with low glucose tolerance [12]. Hence, to fulfill these requirements, much research has modified starch with chemical, physical, and enzymatic techniques [13] to provide unique starch characteristics with better thermal stability, mechanical stability [14,15,16], and digestibility [17].

Heat-moisture treatment (HMT) is one of the widely used approaches to physically modifying the properties of starch [18]. The advantages of HMT include fewer toxic effects, and the procedure is generally considered as safe. Furthermore, several studies have shown that HMT provides significant benefits to physicochemical properties, rheological properties, and digestibility [19,20,21,22]. Aside from single HMT, other researchers also tested the effect of multiple cycles of HMT and concluded that repeated modifications were capable of enhancing the gelatinization temperature, crystallinity, and digestibility of sweet potato starch [23], wheat starch [24], corn starch, tapioca starch, and potato starch [15,25].

Although single and multiple HMT have exhibited promising effects on physicochemical properties and digestibility in many studies [11,26,27], few studies have examined the effect of cycled heat-moisture treatment on water caltrop starch. A previous study reported that HMT showed significant influence on physicochemical and digestibility of water caltrop starch [28]. Therefore, we assumed that cycled heat-moisture treatment may exert more pronounced effects on water caltrop starch in comparison to a single cycle of HMT. To examine our hypothesis, we tested the effect of cycled heat-moisture treatment on the in vitro digestibility, rheological properties, and physicochemical properties of water caltrop starch.

## 2. Materials and Methods

### 2.1. Materials

Water caltrops (*Trapa taiwanensis* Nakai) were cultivated and harvested in Tainan, Taiwan. The α-amylase, amyloglucosidase, amylose kit, and damaged starch kit were purchased from Megazyme (Megazyme International Ireland, Co. Wicklow, Ireland). All chemical substances applied in this study were of analytical grade.

### 2.2. Starch Isolation

The extraction of starch was applied in accordance with a previous report with slight modifications [29]. In the typical extraction, the fresh kernels of water caltrop were blended with twice the volume of distilled water and mixed with 0.2% (*w*/*v*) sodium hydroxide solution. The mixture was placed at 4 °C for 12 h; thereafter, the upper brown liquid was decanted, and the starch precipitate was washed with distilled water and left to precipitate. The washing process was repeated until the starch was clean and the pH value of starch dispersion reached neutral. After removing the impurities, the starch pellet was dried at 40 °C for 72 h, ground, passed through a 100-mesh sieve, and stored at room temperature. The unmodified starch was designated as Native-S.

### 2.3. Cycled Heat Moisture Treatment

A previous method was applied including some modifications [30]. The moisture level of water caltrop starch was adjusted to 20% by adding adequate distilled water. The mixtures were stirred, sealed in the tinplate cans, and modified by cycled heat-moisture treatment (cHMT) at 105 °C with different cycles. The total heating time for each sample was 16 h; however, the heating time was divided into 1 cycle (16 h/cycle), 2 cycles (8 h/cycle), 4 cycles (4 h/cycle), and 8 cycles (2 h/cycle), with 30 min of cooling at room temperature between each heating procedure. The cycling numbers of 1, 2, 4, and 8 were designated as cHMT1, cHMT2, cHMT4, and cHMT8, respectively. The modified samples were dried at 40 °C until the moisture level reached approximately 10% then milled and screened through a 100-mesh sieve and stored at room temperature.

### 2.4. Amylose and Damaged Starch Content

The amylose and damaged starch content of water caltrop starch was determined by using a Megazyme amylose kit (K-AMYL) and a damaged starch kit (K-SDAM), respectively, with the procedures recommended by the manufacturer.

### 2.5. Molecular Weight Distribution

The molecular weight distribution profile of water caltrop starch was evaluated by gel permeation chromatography (GPC) using a column (2.6 × 90 cm diameter/height) packed with Sepharose CL-2B agarose gel. Sample preparation was according to a method from previous research [31]. A 5 mL aliquot including 15 mg of starch and glucose marker was injected in the column and underwent elution in the ascending mode. The eluting buffer contained 25 mM NaCl, 1 mM NaOH, and 0.02% NaN3 and had a flow rate of 30 mL/h. Fractions of 100 tubes were collected (5 mL/tube), and total sugar content was measured using the phenol-sulfuric acid method at 490 nm, while iodine-stained blue value was measured at 630 nm. The standard curve was made of dextran with different molecular weights prepared with a similar method to that mentioned above. The weight-average molecular weight was calculated:

(1) $$\bar{\mathrm{Mw}}=\frac{\Sigma \mathrm{Mi}\;\times \;\mathrm{Ci}}{\Sigma \mathrm{Ci}}$$

In Equation (1), Mi is the molecular weight at ith fraction, and Ci is the glucose concentration at ith fraction (μg/mL).

### 2.6. Thermal Properties

Thermal properties of water caltrop starch were measured by using differential scanning calorimetry (DSC 1 STAR system, Mettler Toledo, Switzerland) according to the previous method [19]. Samples were prepared by mixing 10 mg of starch with 40 mg of pure water. The mixtures were then vigorously shaken to disperse the starch evenly. Then, 6 mg of the mixture was instantly transferred into an aluminum crucible using a pipette. The crucibles were sealed hermetically and stored at 4 °C for 24 h before analysis. The measurement involved heating from 25 to 95 °C at a rate of 10 °C/min. Then, the onset temperature (T_0_), peak temperature (T_p_), conclusion temperature (T_c_), and gelatinization enthalpy (ΔH) were recorded during the heating process.

### 2.7. Steady Shear Rheological Measurement

Steady shear properties were measured according to the previous method [32] with a rheometer (MCR92, Anton Paar, Graz, Austria). The starch paste (6%, *w*/*w*) was prepared by using RVA (RVA-Ezi, Newport Scientific Pty. Ltd., Warriewood, Austria) at a rotation speed of 160 rpm, heated from 50 to 95 °C for 3.5 min, and held at 95 °C for 3 min for full gelatinization. Then, the starch sample was rapidly poured into a rotational concentric cylinders, equilibrated at 25 °C for 3 min before analysis, and continuously sheared in the gap (0.099 mm) between the inner cylinder (radius: 26.652 mm, length: 39.999 mm) and the outer cylinder (radius: 28.922 mm) from shear rate of 1 to 100 s^−1^. Herschel–Bulkley model was applied to describe the flow behavior of the samples at steady shear:

(2) $$\sigma =\sigma_{0}\;+\;K{\left(\overset{\cdot }{\gamma }\right)}^{n}$$

In Equation (2), σ is the shear stress (Pa), σ_0_ is the yield stress (Pa), K is the consistency index (Pa.s^n^), $\overset{\cdot }{\gamma }$ is the shear rate (s^−1^), and *n* is the flow behavior index (dimensionless).

### 2.8. Dynamic Rheological Properties

The dynamic properties of water caltrop starch were measured using the same rheometer equipped with a parallel plate (diameter: 50.00 mm) and with a 1 mm gap using the previous method with slight modifications [32]. Meanwhile, the samples were prepared using RVA similar to the method described in steady shear measurement. The starch paste was sheared in the range of 0.1 to 100 rad/s at 1% strain, which was in the linear viscoelastic region. The storage modulus (G′), loss modulus (G″), and loss tangent (tan 𝛿) were measured at 25 °C.

### 2.9. In Vitro Digestibility and Predicted Glycemic Index of Cooked Starch

The in vitro kinetics and predicted glycemic index were inspected with the method reported previously [33,34] with slight modifications. Starch samples (100 mg, dry basis) were mixed with 2.5 mL of distilled water and cooked in boiling water for 10 min with continuous stirring by magnetic bars. Then, 2 mL of sodium maleate buffer (100 mM, pH 6.0 with 2 mM calcium chloride) was immediately added, and samples were incubated at 37 °C for 5 min in a shaking water bath. Next, 2 mL of pancreatic α-amylase (93.75 Ceralpha unit/mL) and amyloglucosidase (9.375 U/mL) was added and incubated at 37 °C with shaking (200 rpm) for digestion. Aliquots of 0.08 mL hydrolyzed solution were taken at 5, 10, 20, 40, 80, 120, and 180 min, respectively. The aliquots were mixed with 0.64 mL of 95% ethanol solution to inactivate the enzymes. The glucose content of 0.1 mL hydrolysates was evaluated by a Megazyme D-Glucose Assay Kit (GOPOD) containing glucose oxidase and peroxidase solution. The hydrolysis percentage was calculated as described:

(3) $$\frac{\mathrm{Gt}\;\times \;\frac{0.72}{0.1}\;\times \;\frac{6.5}{0.08}{\;\times \;10}^{-3}\;\times \;0.9}{100}\;\times \;100$$

In Equation (3), Gt is the glucose concentration (μg/0.1 mL) at time t. The curve of enzyme hydrolysis followed a first-order equation. For reference, dried white toast was used. The calculation of predicted glycemic index followed the equations:

(4) $${\mathrm{HP}=\mathrm{HP}}_{\infty }\;\times \;\left({1-e}^{-\mathrm{kt}}\right){,\;\mathrm{HP}}_{\infty }\leq 100\%$$

(5) $$\mathrm{Area}\;\mathrm{under}\;\mathrm{curve}\;\left(\mathrm{AUC}\right)=\int_{0}^{180}{\mathrm{HP}}_{\infty }\;\times \left({1-e}^{-\mathrm{kt}}\right)\mathrm{dt}$$

(6) $$\mathrm{Hydrolysis}\;\mathrm{index}\;\left(\mathrm{HI}\right)=\frac{{\mathrm{AUC}\;}_{\mathrm{samples}}}{{\mathrm{AUC}\;}_{\mathrm{reference}}}$$

(7) Predicted glycemic index (pGI)=39.71+0.549 × (HI)

In Equations (4)–(7), HP is the hydrolysis percentage at time t, HP_∞_ is the equilibrium hydrolysis percentage, k is the kinetic constant, t is the chosen time, AUC_samples_ is the area under curve of samples, and AUC_reference_ is the area under curve of the reference white bread.

### 2.10. Statistical Analysis

The measurements were performed in triplicate, and the data were analyzed with one-way analysis of variance (ANOVA) followed by Duncan’s multiple range test with SPSS 20.0 at a significance level of 95% (*p* < 0.05).

## 3. Results and Discussion

### 3.1. Amylose and Damaged Starch Content

As shown in Table 1, the amylose content was significantly influenced by cHMT. Native water caltrop starch (Native-S) contained 25.01% amylose, which is similar to previous results for water caltrop starch [1,35]. However, after modification, the amylose content significantly increased, and this can be explained by the breakage of side chains of amylopectin [36]. Heat-moisture treatment provided energy to cleave the α-1,6 glycosidic bond in the amorphous regions and caused the sides chains of amylopectin to break and turn into small fractions of amylose and increase the amylose content.

The damaged starch content of water caltrop starch showed no significant difference at Native-S, cHMT1-S, and cHMT2-S. However, when more cycles were applied (cHMT4-S and cHMT8-S), the damaged starch content significantly lowered. This was possibly due to the accelerated recrystallization during cHMT, which enhanced the resistance to the tested enzymes and reduced the damaged starch content [37]. These results were in line with other studies on mung bean starch, potato starch, corn starch, and waxy corn starch, which showed reduced damaged starch content after heat-moisture treatment [38].

### 3.2. Molecular Weight Distribution

**Figure 1:**
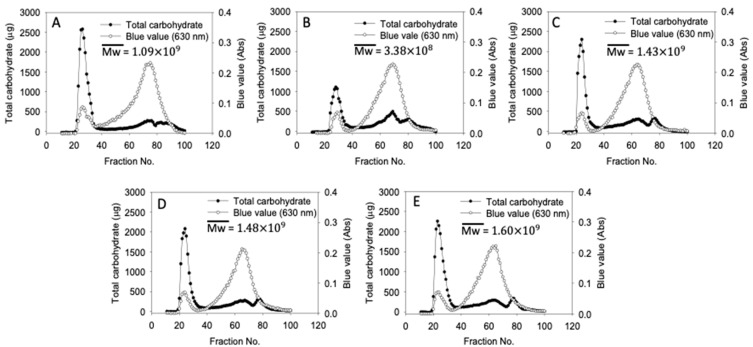
Starch molecular size distribution of water caltrop starch. (**A**) Native-S, (**B**) cHMT1-S, (**C**) cHMT2-S, (**D**) cHMT4-S, (**E**) cHMT8-S.

Gel permeation chromatograms of native and cHMT water caltrop starch are presented in Figure 1. The first peak correlated to amylopectin, the second peak with considerable blue value correlated to amylose, and the last peak was the glucose marker. In cHMT1-S, the peak of amylopectin apparently decreased, resulting in lower weight-average molecular weight. The reduction of the amylopectin peak was attributed to the excessive heat provided by the single cycle of modification, which disrupted the double helices in the amorphous region and broke the sidechain of amylopectin into small fractions of amylose [39]. A previous report on heat-moisture treated breadfruits showed similar reductions in amylopectin peak and weight-average molecular weight with a heat-moisture treatment [30]. However, in cHMT2-S, cHMT4-S, and cHMT8-S (multiple cycles applied), the weight-average molecular weight increased up to 1.60 $\times \;10^{9}$. This may be explained by enhanced retrogradation of starch molecules in repeated cooling procedures, which prompted the interaction of amylopectin [40]. Hence, amylopectin with larger molecular weight was increased and led to higher weight-average molecular weight [41,42].

### 3.3. Thermal Properties

As shown in Table 2, the thermal properties of water caltrop starch were changed significantly by cHMT. Higher T_0_, T_p_, and T_c_ were observed in cHMT water caltrop starch; this was possibly related to higher amylose content, which resulted in more interactions between the starch molecules. Therefore, a higher temperature would be needed to disrupt the order of starch granules [43]. cHMT included repeated cycles of heating and cooling, which facilitated the rearrangement of starch molecules. As a result, more compact structures were formed and hindered the transfer of heat during DSC examination. These results were similar to the behavior of multi-cycled heat-moisture treated azuki bean starch [44], sweet potato starch [23], and wheat starch [45]. cHMT2-S, cHMT4-S, and cHMT8-S with more than one cycle of cHMT showed higher and similar T_p_ with no significant difference, indicating their similarity and better heat stability after cHMT. The decrement of ΔH may be due to lower amylopectin content, which contributed to fewer double helices inside the crystalline domain and lower enthalpy acquired for gelatinization [46].

### 3.4. Steady Shear Rheological Measurement

**Figure 2:**
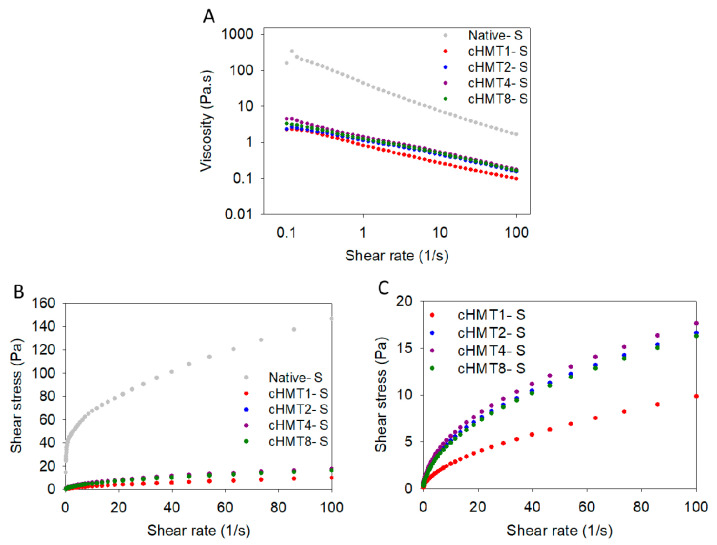
Steady shear rheological properties of native and cHMT water caltrop starch paste. (**A**) Viscosity versus shear rate. (**B**) Shear stress versus shear rate of native and cHMT starch. (**C**) Shear stress versus shear rate of cHMT starch.

The curves of steady shear measurement of native and cHMT water caltrop starch pastes are presented in Figure 2. The viscosity decreased with increasing shear rate; this implied that water caltrop starch paste behaved as a non-Newtonian fluid. A similar result was also found in Kunth root starch [47].

The parameters of the Herschel–Bulkley model used to describe the flow curves are presented in Table 3. It was found that the determination coefficients (R^2^) of each sample ranged from 0.968 to 0.999, suggesting that the Herschel–Bulkley model fitted well to the profile of rheological characteristics. The flow behavior index (*n*) reflects the similarity to Newtonian fluid; *n* = 1 equals Newtonian fluid and a lower value of *n* indicates a higher degree of shear-thinning properties. Both native and cHMT samples showed *n* values of less than 1.0, implying that water caltrop starch pastes exhibited shear-thinning behavior. Furthermore, with increasing cycles of cHMT, the *n* value also increased progressively, indicating that cHMT samples showed less shear-thinning behavior. The K value and σ_0_ could be considered as the gel viscosity and stress to initiate the flow, respectively [48]. Compared to Native-S, modified water caltrop starch showed lower values of K and σ_0_, indicating that modified starch had lower viscosity and required less stress to start the flow.

### 3.5. Dynamic Rheological Properties

**Figure 3:**
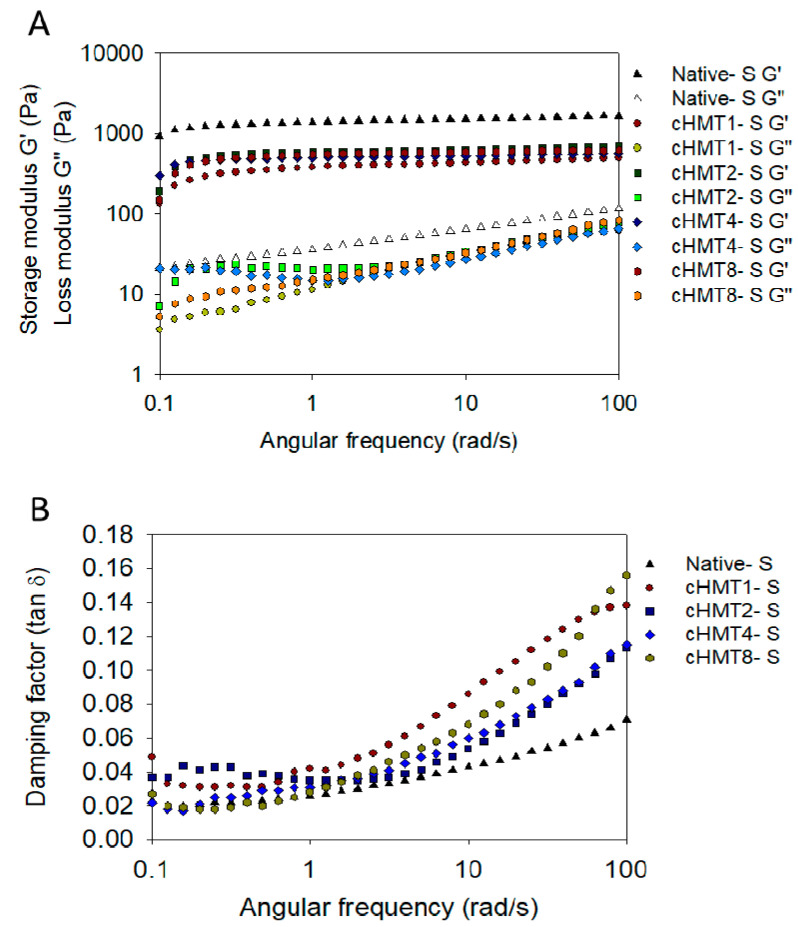
Dynamic rheological behavior of native and cHMT water caltrop starch. (**A**) Angular frequency dependence of G′ and G″ at 25 °C for starches; (**B**) angular frequency dependence of tan 𝛿 at 25 °C for starches.

The storage modulus (G′) is the amount of deformation energy stored in the sample during shear, and it represents the elasticity of the sample. In contrast, the loss modulus (G″) is the amount of deformation energy consumed during shear and represents the viscosity of the sample [48]. The ratio of G″/G′ is called the loss tangent (tan 𝛿), which can identify the viscoelastic properties of the gel. As shown in Figure 3, the storage modulus (G′) was significantly higher than the loss modulus (G″) in water caltrop starch and indicated that all samples show solid-like characteristics.

After modification, the G′ and G″ values were gradually reduced as the number of cycles decreased, and the parameters are shown in Table 4. This result was similar to the previous findings [48], which reported that heat-moisture treatment encouraged the deformability of starch granules and hindered the formation of strong gel structures. But cHMT8-S, which was treated with the most cycles showed a higher value compared to other groups, indicating the milder modification and better similarity to Native-S.

Tan 𝛿 can be used to describe the characteristics of the gel. When tan 𝛿 is less than 1, the material shows great elasticity; when the value of tan 𝛿 is between 0.1 and 1, it is classified as weak gel; and a tan 𝛿 less than 0.1 shows strong gel characteristics. Tan 𝛿 of water caltrop starch ranged from 0.039 to 0.072, which indicated strong gel properties. Furthermore, cHMT1-S showed the highest value, which may be explained by the strong disruption by a single cycle of cHMT.

### 3.6. In Vitro Digestibility and Predicted Glycemic Index of Cooked Starch

**Figure 4:**
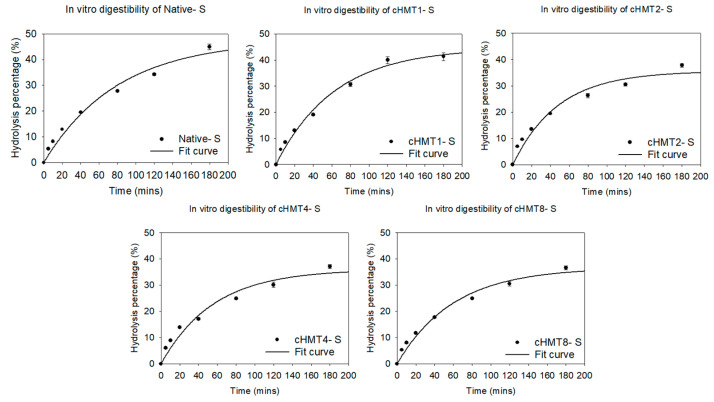
The hydrolysis curve of native and cHMT water caltrop starch.

This experiment used the first-order model to simulate the final hydrolysis percentage (HP_∞_), hydrolysis rate (k), hydrolysis index (HI), and predicted GI value (pGI). The sample hydrolyzed rapidly in the first 60 min and gradually slowed down with the extension of working time (Figure 4).

As shown in Table 5, the HP_∞_ of samples decreased significantly after modification; this may be due to the more ordered structure formed by repeated heating/cooling cycles in cHMT [23]. However, cHMT1-S with a single cycle of treatment had lower HP_∞_, which may be ascribed to strong disruption to double helices and reduced formation of ordered structure, which resulted in more vulnerability to the enzyme.

In terms of digestion rate (k), water caltrop starch increased slightly after modification. cHMT1-S showed greater pGI than Native-S, while the values of cHMT2-S, cHMT4-S, and cHMT8-S were significantly lower than Native-S, indicating that multiple cycles of heat-moisture treatment did reduce the digestibility of water caltrop starch. Lower digestibility may be affected by the amylose content. In previous studies, it was found that starch with a higher proportion of amylose showed lower digestibility and hydrolysis rate because the space occupied by amylose is smaller than that of amylopectin, so the structure of starch could be stacked tighter and not easily digested by enzymes [49,50,51]. In addition, crystalline type and relative crystallinity were also important factors affecting digestibility. Starch with higher relative crystallinity was more compact in structure, so enzymes were less likely to enter the starch granules [52].

## 4. Conclusions

This study showed that cHMT changed the in vitro digestibility, rheological properties, and physicochemical properties of water caltrop starch. The amylose content increased by modification due to the breakage of amylopectin which turned into small amylose fragments. The damaged starch content also significantly decreased in samples treated with an increased number of cycles because of recrystallized structure that expelled the testing enzyme. Single-cycle cHMT decreased the amylopectin peak in the GPC graph and lowered the weight-average molecular weight, but the weight-average molecular weight increased with multiple cycles because repeated cooling in cHMT may have promoted the integration of amylopectin, which had larger molecular weight. Better resistance to the heat and lower enthalpy needed for gelatinization was confirmed by DSC. Furthermore, modification gave water caltrop starch less shear-thinning behavior with higher *n* value and lower gel-forming ability with lower G′ and G″ values. After cooking, cHMT water caltrop starch treated with an increased number of cycles showed lower pGI, which could be explained by the increasing amylose content and more compact structure of starch granules, which led to better resistance to the enzymes. The simulated pGI test may not completely reflect the exact starch digestibility in vivo, and more research was needed to employ cHMT starch as ingredient for application. Such a study would provide a better understanding of the physicochemical properties, digestibility, and potential application of cHMT water caltrop starch, which may be a prospective alternative for common starch resources such as corn and potato.

## Figures and Tables

**Table 1:** Amylose content and damaged starch content of native and cHMT samples ^1,2^.

Treatment	Amylose Content (%, d.b.)	Damaged Starch Content (%, d.b.)
Native-S	25.01 ± 0.70 ^a^	1.26 ± 0.04 ^a^
cHMT1-S	26.16 ± 0.36 ^bc^	1.25 ± 0.01 ^a^
cHMT2-S	25.42 ± 0.61 ^ab^	1.29 ± 0.11 ^a^
cHMT4-S	26.37 ± 0.17 ^c^	0.61 ± 0.06 ^b^
cHMT8-S	26.06 ± 0.20 ^bc^	0.34 ± 0.01 ^c^

^1^ Data presented are in mean ± SD form (*n* = 3); different letters in the same column indicate the significant difference at *p* < 0.05. ^2^ The capital S indicates water caltrop starch, and the number indicates the cycles in cHMT.

**Table 2:** Thermal properties of native/cHMT samples ^1,2^.

Treatment	T_0_ (°C)	T_p_ (°C)	T_c_ (°C)	T_c_–T_0_ (°C)	ΔH (J/g)
Native-S	77.38 ± 0.40 ^a^	81.18 ± 0.10 ^a^	85.77 ± 0.54 ^a^	8.39 ± 0.93 ^a^	4.41± 0.57 ^b^
cHMT1-S	82.61 ± 0.29 ^b^	87.35 ± 0.33 ^b^	94.83± 0.73 ^bc^	12.22 ± 0.44 ^d^	3.35 ± 0.20 ^a^
cHMT2-S	83.66 ± 0.51 ^c^	88.89 ± 0.45 ^c^	95.30 ± 0.33 ^c^	11.34± 0.21 ^cd^	3.39 ± 0.11 ^a^
cHMT4-S	83.21 ± 0.14 ^c^	88.54 ± 0.09 ^c^	94.41 ± 0.08 ^b^	11.20 ± 0.18 ^c^	3.74 ± 0.05 ^a^
cHMT8-S	84.32 ± 0.18 ^d^	88.69 ± 0.20 ^c^	94.32 ± 0.17 ^b^	10.00 ± 0.12 ^b^	3.76 ± 0.06 ^a^

^1^ Data presented are in mean ± SD form (*n* = 3); different letters in the same column indicate the significant difference at *p* < 0.05. ^2^ The capital S indicates water caltrop starch, and the number indicates the cycles in cHMT.

**Table 3:** Herschel–Bulkley model parameters of native and cHMT water caltrop starch ^1,2^.

Treatment	σ_0_ (Pa)	*n*	K (Pa.s^n^)	R^2^
Native-S	17.290 ± 0.880 ^b^	0.380 ± 0.016 ^a^	21.006 ± 0.885 ^b^	0.968
cHMT1-S	0.032 ± 0.018 ^a^	0.569 ± 0.072 ^b^	1.466 ± 0.024 ^a^	0.994
cHMT2-S	0.196 ± 0.036 ^a^	0.604 ± 0.020 ^b^	1.178 ± 0.036 ^a^	0.995
cHMT4-S	0.237 ± 0.068 ^a^	0.647 ± 0.022 ^c^	1.099 ± 0.107 ^a^	0.993
cHMT8-S	0.081 ± 0.015 ^a^	0.655 ± 0.029 ^c^	1.131 ± 0.069 ^a^	0.996

^1^ Data presented are in mean ± SD form (*n* = 3); different letters in the same column indicate the significant difference at *p* < 0.05. ^2^ The capital S indicates water caltrop starch, and the number indicates the cycles in cHMT.

**Table 4:** Viscoelastic properties of native and cHMT water caltrop starch at 6.28 rad/s ^1,2^.

Treatment	G’ (Pa)	G’’ (Pa)	tan 𝛿
Native-S	1401.833 ^d^	54.867 ^d^	0.039 ^a^
cHMT1-S	244.087 ^a^	17.365 ^a^	0.072 ^c^
cHMT2-S	458.467 ^b^	19.179 ^b^	0.042 ^ab^
cHMT4-S	589.353 ^c^	30.221 ^c^	0.052 ^ab^
cHMT8-S	626.350 ^c^	34.500 ^c^	0.055 ^b^

^1^ Data presented are in mean ± SD form (*n* = 3); different letters in the same column indicate the significant difference at *p* < 0.05. ^2^ The capital S indicates water caltrop starch, and the number indicates the cycles in cHMT.

**Table 5:** The digestibility of native and cHMT water caltrop starch ^1,2,3^.

Treatment	HP_∞_ (%)	k × 10 ^2^ (min^−1^)	HI	pGI
Native-S	46.13 ± 0.52 ^c^	1.42 ± 0.20 ^a^	79.71 ± 0.53 ^c^	83.47 ± 0.28 ^c^
cHMT1-S	44.58 ± 2.22 ^b^	1.59 ± 0.13 ^ab^	83.29 ± 1.47 ^d^	85.43 ± 0.80 ^d^
cHMT2-S	35.52 ± 0.70 ^a^	2.09 ± 0.01 ^c^	73.44 ± 1.53 ^b^	80.02 ± 0.84 ^b^
cHMT4-S	35.95 ± 1.07 ^a^	1.81 ± 0.01 ^b^	70.69 ± 1.38 ^a^	78.52 ± 0.76 ^a^
cHMT8-S	36.61 ± 1.15 ^a^	1.68 ± 0.01 ^b^	69.70 ± 0.99 ^a^	78.14 ± 0.54 ^a^

^1^ Data presented are in mean ± SD form (*n* = 3); different letters in the same column indicate the significant difference at *p* < 0.05. ^2^ The capital S indicates water caltrop starch, and the number indicates the cycles in cHMT. ^3^ HP_∞_ indicates the equilibrium hydrolysis percentage, k indicates kinetic constant, HI indicates the hydrolysis index, and pGI indicates the predicted glycemic index.

## Data Availability

The data presented in this study are available on request from the corresponding author. The data are not publicly available due to ethical restriction and the intellectual property issue.
